# Fingerprinting sub-basin spatial sediment sources in a large Iranian catchment under dry-land cultivation and rangeland farming: Combining geochemical tracers and weathering indices

**DOI:** 10.1016/j.ejrh.2019.100613

**Published:** 2019-08

**Authors:** Zeinab Mohammadi Raigani, Kazem Nosrati, Adrian L. Collins

**Affiliations:** a Department of Physical Geography, School of Earth Sciences, Shahid Beheshti University, 1983969411, Tehran, Iran; b Sustainable Agriculture Sciences Department, Rothamsted Research, North Wyke, Okehampton, EX20 2SB, UK

**Keywords:** Sediment provenance, Composite fingerprinting, Weathering indices, Dry-land farming, Un-mixing model

## Abstract

**Study region:**

The Kamish River catchment (308 km^2^); a mountainous agricultural catchment under dry-land and rangeland farming located in Kermanshah province, in western Iran.

**Study focus:**

The main objective of this study was to apportion sub-basin spatial source relative contributions to target channel bed sediment samples using a composite fingerprinting procedure including a Bayesian un-mixing model. In total, thirty-four geochemical tracers, eleven elemental ratios and different weathering indices were measured or estimated for 43 tributary sediment samples collected to characterise three sub-basin spatial sediment sources and eleven target bed sediment samples collected at the outlet of the main basin. Statistical analysis was used to select three different composite signatures.

**New hydrological insights for the region:**

Using a composite signature based on KW-H and DFA, the respective relative contributions (with uncertainty ranges) from tributary sub-basins 1, 2 and 3 were estimated as 54.3% (47.8–62.0), 11.4% (4.2–18.7) and 34.3% (27.6–39.9), compared to 72.0% (61.6–82.7), 13.6% (9.0–18.5) and 14.2% (3.1–25.4) using a combination of KW-H and data mining, and 50.8% (42.8–59.9), 28.7% (20.2–37.3) and 20.3% (12.7–27.2) using a fingerprint selected by KW-H and PCCA. The root mean square difference between these source estimates highlighted sensitivity to the composite signatures. Evaluation of the un-mixing model predictions using virtual mixture tests confirmed agreement between modelled and known source proportions.

## Highlights

• Previous studies suggest source tracing should test different types of tracers.
• Geochemical tracers were tested together with lithological weathering indices.
• Composite fingerprints apportioned sub-basin spatial source contributions.
• The findings reveal the sensitivity of source apportionment estimates to the fingerprints.
• Virtual mixture tests evaluated the un-mixing model predictions.

## Graphical abstract

**Figure:**
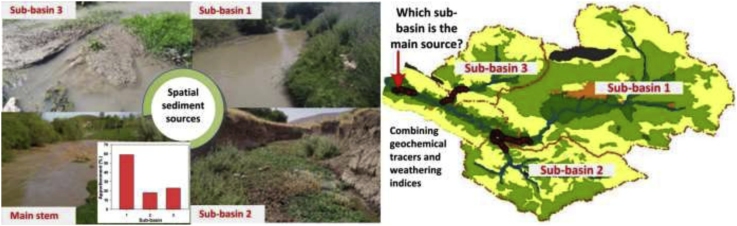

## 1 Introduction

Accelerated soil erosion is a serious environmental problem (Wang et al., 2018) affecting the physical, chemical and biological quality of land and water worldwide (Malhotra et al., 2018). Although fine-grained sediment is a naturally occurring material in river systems, providing a fundamental input for the development of river landscapes and their aquatic ecosystems (Frostick et al., 1984; Lisle, 1989), excessive fine-grained sediment delivery to, and accumulation within, river systems is one of the most pervasive causes of watercourse degradation worldwide (Gellis and Gorman Sanisaca, 2018). In fact, since the 1980′s there has been growing concern in many countries over the off-site issues associated with accelerated soil erosion (Evans et al., 2017), including muddy floods and the related damage to properties (Boardman et al., 1996), increased operational and maintenance costs for potable water purification (Cashman et al., 2018; Owens et al., 2016), deformation of channel morphology (Cashman et al., 2018; Collins and Walling, 2007; Gellis and Gorman Sanisaca, 2018; Haddadchi et al., 2013; Zhou et al., 2016), the excessive destruction of the earth (Wang et al., 2018), the transfer and redistribution of nutrients and pollutants (Blake et al., 2018; Carter et al., 2003; Collins and Walling, 2007; Westrich and Förstner, 2007), and the harmful effects on aquatic biology (Jones et al., 2014, 2012; Kemp et al., 2011; Wilkes et al., 2019). Alongside these off-site impacts, a wide range of on-site problems also result from accelerated soil erosion and sediment mobilization including, amongst others, reductions in soil fertility and water holding capacity and reduced crop productivity (Nosrati, 2017; Tiecher et al., 2018).

Against the above context, accelerated soil erosion is rated as a serious threat to sustainable land and water resource management in many parts of the world and, in turn, this has driven a growing awareness of the need to understand the contributions of different sediment sources and erosion processes to sediment-related problems on- and off-site (Froger et al., 2018; Laceby et al., 2017). Improved understanding of sediment sources can be utilized as an important basis for the conceptualization and confirmation of the drivers (e.g. modern intensive farming) of accelerated soil erosion and sediment transport (Gellis and Gorman Sanisaca, 2018; Walling et al., 1993; Wang et al., 2018; Zhou et al., 2016), and to help target management (Laceby et al., 2017; Walling, 2005). To this end, uptake and refinement of sediment source tracing or fingerprinting techniques has expanded dramatically as an alternative approach to traditional methods of identifying key sediment sources (Blake et al., 2018; Carter et al., 2003; Collins et al., 1998, 2017b; Collins and Walling, 2007; Walling et al., 1993, 1999) and erosion processes in many environmental settings (Collins et al., 2010, 2017a; Froger et al., 2018; Haddadchi et al., 2013; Koiter et al., 2013). Sediment source fingerprinting involves discriminating potential sediment sources on the basis of differences in source material properties or tracers and determining the relative contributions of these sources to sampled target sediment.

A wide range of tracer properties has been tested for the identification of sediment sources (Bainbridge et al., 2016; Chen et al., 2017; Collins and Walling, 2002; Collins et al., 2017b; Estrany et al., 2010; Grimshaw and Lewin, 1980; Haddadchi et al., 2013; Hatfield and Maher, 2009; Mckinley et al., 2013; Nosrati et al., 2018b; Palazón and Navas, 2017; Walling et al., 1999). Geochemical tracers are, however, the most widely used in published studies to date (D’Haen et al., 2012; Froger et al., 2018; Haddadchi et al., 2014, 2013; Hughes et al., 2009; Koiter et al., 2013; Nosrati et al., 2018b; Olley and Caitcheon, 2000) because of their frequent ability to discriminate strongly among different sources and to apportion sources of fine-grained sediment with reasonable levels of uncertainty (Malhotra et al., 2018). The use of one single tracer to apportion sources is inappropriate given the inherent risk of spurious source-sediment matches (Carter et al., 2003; Collins et al., 1998; Malhotra et al., 2018; Mukundan et al., 2012; Walling et al., 1999; Zhou et al., 2016). In response, so-called composite fingerprinting approaches have been developed based on combining several tracer properties into fingerprints. The use of such “composite signatures” provides a more reliable and consistent means for determining sediment sources (Walling et al., 1999), reduces the likelihood of false source-sediment links and often provides the scope for discriminating more sources (Collins et al., 1998, 2017b).

In this study, a composite fingerprinting approach, using statistical validation of different composite signatures, and a multivariate Bayesian un-mixing model, was applied for determining the relative contribution of each of three sub-basins of the Kamish River, in Iran, to downstream target bed sediment samples collected on the main stem. Land managers and policy makers frequently seek information on the contributions of sediment from individual source types, classified for example, on the basis of generic land use categories such as arable or grass, but also including additional sources such as channel banks, since this type of source classification scheme provides a clear means of selecting relevant mitigation measures capable of reducing sediment mobilization associated with tillage methods, livestock management or fluvial scour of channel margins. In larger catchments, however, where land use patterns can be complicated, and the contributions from individual source types more spatially variable, provision of such information exclusive of some form of underpinning spatial information on the general sources of sediment problems, is not necessarily supportive of decision making which is commonly based on limited resources for on-the-ground follow-up and intervention planning. Here, previous work applying sediment fingerprinting procedures has adopted an alternative approach to the classification of potential sources, based on tributary sub-catchments (e.g., Caitcheon, 1993; Collins et al., 1997; Hardy et al., 2010; Nosrati et al., 2018a; Stone et al., 2014; Vale et al., 2016; Wilkinson et al., 2013). In essence, this confluence-based approach permits investigation of the sediment contributions from different spatial zones within a large catchment, and thereby a tier 1 screening of sediment sources for guiding the targeting of any future investigations into individual source types. The work reported herein thereby summarizes the first pass screening of sediment sources in the study catchment and was designed to provide data on the contributions from spatial zones represented by tributary sub-catchments.

## 2 Materials and methods

### 2.1 Study area

**Fig. 1:**
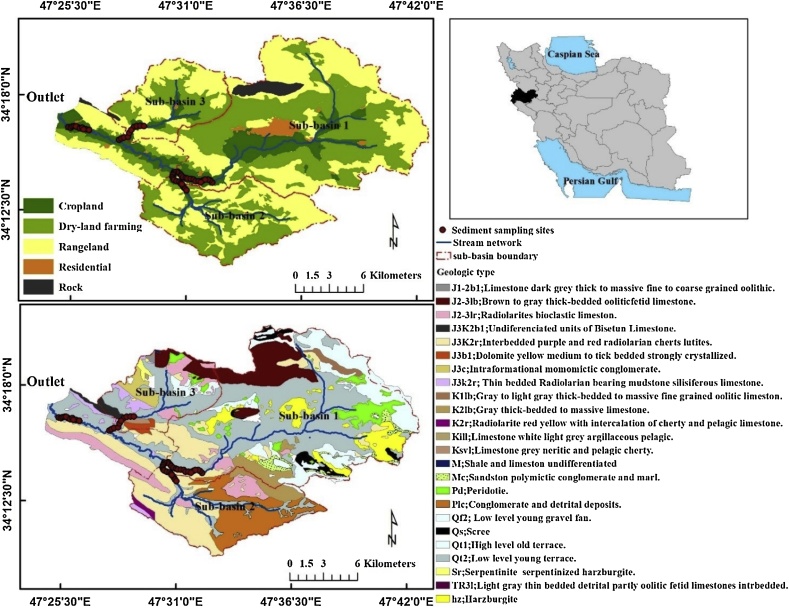
Location map showing the Kamish River catchment in Iran, bed sediment sampling locations in the three tributary sub-basins and downstream on the main stem, and the main geological subdivisions of the study area.

The Kamish River catchment (308 km^2^) is located near the town of Kermanshah province, in western Iran between 47° 24′E to 47° 42′E longitude and 34° 9′N to 34° 20′N latitude (Fig. 1). The study area is one of the branches of the Gamasyab River which, in turn, is located in the drainage basin of the Seymareh River. The topography of the Kamish River catchment is mountainous, with elevations ranging from 1300 to 2600 m, with a mean of 1900 m above sea level. The average slope gradient is 11% and the main river slope is 1.8%. Land cover comprises rangelands (133 km^2^; 43%), cultivated land including dry-land farming, cropped fields and horticulture (163 km^2^; 52.9%), rock outcrops (6 km^2^; 1.9%) and residential areas (6 km^2^; 1.9%).

Long-term (30 years) mean annual precipitation recorded at the Kamish station next to the study area is ca. 370 mm. In the upper parts of the region, precipitation is mostly snow. Mean annual discharge based on the 30 years (1985–2015) of record from the hydraulic station at the outlet of the drainage basin is estimated at 1.21 m^3^ s^−1^, with most discharge occurring in March and April: monthly means of 11 m^3^ s^−1^ and 5.81 m^3^ s^−1^, respectively. On the basis of 10 field surveys and associated observations over a 12-month period, it was established that the three sub-basins (Fig. 1) comprising sub-basin 1 (168.3 km^2^), sub-basin 2 (63.7 km^2^) and sub-basin 3 (43 km^2^) in the study area dominate tributary sediment inputs to the main river. Land use in the three sub-basins is summarized in Table S1 in Supplementary Information.

**Fig. 2:**
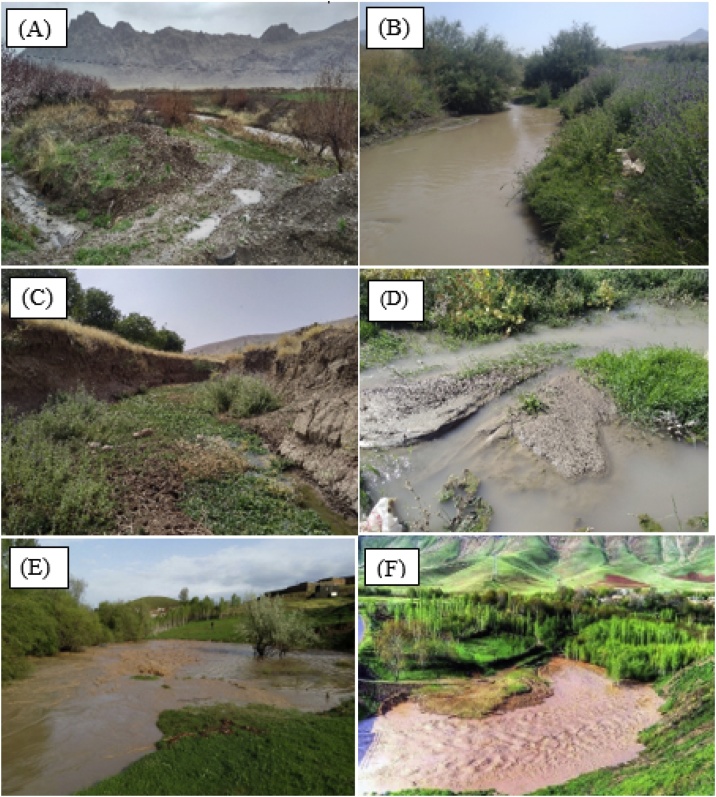
Photos taken at the outlets of (A) the main catchment (target sediment sampling location for apportioning sub-basin sources) and three upstream sub-basins (representing potential spatial sediment sources): (B) sub-basin 1, (C) sub-basin 2, and (D) sub-basin 3. Photos showing the off-site impacts of sediment mobilisation and delivery on (E) gardens and (F) agricultural land in the Kamish River catchment.

From a stratigraphical point of view (Fig. 1), ancient geological formations belonging to the Precambrian period and more recent formations belonging to the Quaternary period are observed in the study area (see Table S2 in Supplementary Information). Based on field observations, erosion is very evident across the study area. After any rainfall event, a large volume of soil undergoes erosion and subsequent transport and is deposited on the main stem channel bed at the basin outlet. The average daily suspended sediment export has been estimated at 3814 tons (Iran Water Resources Management Company). Runoff and erosion has important off-site impacts in this drainage basin with, for example, muddy floods affecting crop fields and gardens (Fig. 2). As a result, considerable areas of the gardens and agricultural land surrounding the river have been affected by sediment mobilization and delivery, leading to social and economic problems in the locality. Measures for controlling runoff such as terracing, strip cropping and improved vegetation cover are still lacking.

The Kamish River catchment has at its center Harsin City and it is also home to a large number of villages mostly located alongside the banks of the Kamish River. Most villagers in this area are heavily dependent on agriculture, gardening and animal husbandry as their main sources of income. Hence, soil protection in this area is critical for the sustainability of the villages and the wellbeing of villagers. At the present time, the Kamish dam (4.5 km to the south of Harsin City and about 1.5 km to the north of Baba Zeyd village) with a storage capacity of 16.6 million m^3^ and an average annual input flow of 20.5 million m^3^ is being constructed. Current high levels of erosion and sediment production in the region have the potential to impact on the water storage capacity behind the dam. Accordingly, appraisal of the spatial origins of sediment are very important for controlling and managing this risk to new critical infrastructure for supporting livelihoods and well-being.

### 2.2 Field sampling

Although most sediment fingerprinting studies have relied on sets of suspended sediment samples from various flood events or different times across flood hydrographs (Haddadchi et al., 2014; Hughes et al., 2009), fine-grained sediment deposited on the river bed has also been successfully used as an alternative type of target sediment in various environmental situations (Dirszowsky, 2004; Evrard et al., 2011, 2013; Haddadchi et al., 2014, 2013; Hughes et al., 2009; Lamba et al., 2015; Nosrati et al., 2018a; Olley and Caitcheon, 2000; Smith and Blake, 2014). This type of target fine-grained sediment, which has been referred to in some previous studies as “drape” sediment (Nosrati et al., 2018a), can be assumed to provide a surrogate for suspended sediment-associated geochemistry (Horowitz et al., 2012) and to be more temporally representative than snapshot instantaneous suspended sediment samples (Zhou et al., 2016), whilst negating the need for water sample collection during actual storm periods (Miller et al., 2015). On this basis, samples of sediment deposited on the river bed were collected at the outlet of the main drainage basin and from the outlets of the three sub-basins (Fig. 1). Here, the samples collected from the main basin are used as the target sediment for apportioning the relative contributions from the upstream sub-basins (Nosrati et al., 2018a). Each bed sediment sample was a combination of 5 sub-samples each of which was taken at an interval of 200 m along the selected reach. All samples were collected using a non-metallic shovel to prevent sample contamination (Franz et al., 2013) and using a portable stilling well to avoid the winnowing of fines during sample extraction through the water overlying the channel bed. The inclusion of sub-samples was aimed at taking some account of the local scale spatial variation in bed sediment tracer properties. In total, 10 composite samples were retrieved from the main basin outlet and a further 43 composite samples from the ​​three sub-basin outlet sampling locations. The mass of each composite bed sediment sample was ˜1 kg. Due to possible problems associated with the erosion and collapse of channel banks proximal to the bed sediment sampling points, and the risk of bias in the bed-sediment associated geochemistry (Zhou et al., 2016), the downstream reaches on either the sub-basins or on the main stem were pinpointed to channel sections where local bank erosion was not evident.

### 2.3 Laboratory measurements of tracers and estimation of weathering indices

All sediment samples retrieved from the river bed were slowly air-dried and then manually disaggregated using a mortar and pestle. The samples were dry sieved using a <63 μm sieve prior to laboratory analysis in order to compare a similar particle size fraction of all samples (Palazón and Navas, 2017; Pulley et al., 2015; Tiecher et al., 2018). Selection of this size fraction is commonly applied to help reduce the potential bias related to the effects of grain size (Collins et al., 1997; D’Haen et al., 2012; Dirszowsky, 2004; Fu et al., 2008; Manjoro et al., 2017; Nosrati et al., 2011, 2014; Peart and Walling, 1986; Tiecher et al., 2018; Walling et al., 1999), which could directly distort comparison of the sub-basin source and target sediment samples (Nosrati et al., 2018a). Sieving and weighing of the resulting fractions of the sediment samples collected from both the tributaries and the downstream main outlet confirmed it was appropriate to use the <63 μm fraction.

In order to measure the concentration of geochemical tracers including Ag, Ar, As, Ba, Be, Ca, Cd, Ce, Co, Cr, Cu, Fe, K, La, Li, Mg, Mn, Mo, Na, Ni, P, Pb, S, Sb, Sc, Sr, Th, Ti, U, V, Y, Ya, Zn and Zr using an Agilent (Varian) 735 Inductively Coupled Plasma Optical Emission Spectrometer (ICP-OES), 0.25 g of each sediment sample (<63 μm) was digested with hydrofluoric acid (HF) and aqua regia (HCl–HNO_3_; 3:1) at 220 °C for 4 h in a hot box. The concentrations were expressed in milligrams per kilogram. The results showed that the analytical error for each element was <5%. In addition to the 34 elements, several elemental ratios (Al/Ti, Cu/Pb, Fe/Mn, Fe/P, K/Na, Pb/Ni, Al/(K + Na + Ca + Mg), the Chemical Index of Alteration (CIA), the Weathering Index of Parker (WIP), the Chemical Index of Weathering (CIW), the Plagioclase Index of Alteration (PIA), and a recycling ratio (the ratio between the CIA and the WIP) was also computed for potential use in the composite signatures. Table 1 presents a summary of the laboratory results, elemental ratios and weathering indices.

**Table 1:** Tracer concentration data for the different sub-basin spatial sediment sources and downstream target sediment samples, conservation test results, and the Kruskal-Wallis H-test results for discriminating the sub-basin spatial sediment sources. The units of all elements are mg kg-1.

Tracer	Spatial sediment sources						Kruskal- Wallis H-test		Sediment samples (n = 11)	
	Sub-basin 1 (n = 18)		Sub-basin 2 (n = 14)		Sub-basin 3 (n = 10)					
	Mean	SD	Mean	SD	Mean	SD	H value	p-value	Mean	SD
Ag	0.30	0.04	0.31	0.04	0.32	0.03	n.c.	n.c.	0.31	0.06
Al	34308.8	2309.3	40187.9	2361.9	38521.2	1809.1	25.9	< 0.001*	34860.6	1396.8
As	2.9	0.27	3.9	0.60	3.3	0.24	27.8	< 0.001*	3.0	0.18
Ba	190.4	7.7	227.3	10.3	184.2	5.1	30.3	< 0.001*	192.0	6.8
Be	1.0	0.11	1.3	0.09	1.1	0.07	23.2	< 0.001*	1.1	0.06
Ca	122656.1	6081.6	109483.2	6260.6	108516.9	5898.1	23.5	< 0.001*	116793.2	5502.5
Cd	0.28	0.02	0.30	0.01	0.29	0.03	10.6	< 0.001*	0.28	0.01
Ce	42.3	2.9	45.1	1.5	41.6	1.3	n.c.	n.c.	40.3	1.8
Co	21.6	4.1	14.8	0.80	20.4	0.70	27.8	< 0.001*	16.6	1.4
Cr	417.3	331.6	147.5	18.4	248.5	21.7	28.7	< 0.001*	212.6	31.2
Cu	31.4	3.8	30.0	2.4	30.0	0.82	n.c.	n.c.	26.1	1.7
Fe	28973.4	2621.4	26370.2	1449.4	31536.5	1404.3	21.3	< 0.001*	26510.1	1129.5
K	9490.3	877.4	11346.8	627.4	9464.7	261.4	27.3	< 0.001*	9636.6	431.5
La	19.1	1.3	19.6	0.65	17.9	0.57	n.c.	n.c.	17.6	0.8
Li	21.4	2.1	21.5	1.6	19.3	0.67	14.4	< 0.001*	20.9	0.94
Mg	21781.6	2772.4	14292.6	1234.0	19265.9	420.7	32.1	< 0.001*	18161.2	940.7
Mn	604.1	56.5	724.7	63.9	624.1	15.6	n.c.	n.c.	524.4	46.0
Mo	0.66	0.04	0.7	0.04	0.68	0.05	12.3	0.002*	0.66	0.05
Na	4505.8	198.5	4194.5	391.4	3774.8	315.3	24.0	< 0.001*	3756.2	356.8
Ni	222.9	60.1	88.9	11.2	174.5	7.8	32.0	< 0.001*	157.8	15.3
P	795.9	47.1	656.6	43.4	858.6	67.0	29.6	< 0.001*	779.5	45.3
Pb	11.2	1.6	11.6	1.5	11.4	1.1	n.c.	n.c.	9.4	1.6
S	689.3	545.5	369.0	52.2	407.0	98.3	18.9	< 0.001*	423.0	80.4
Sb	1.0	0.12	1.0	0.03	1.0	0.06	n.c.	n.c.	1.0	0.06
Sc	9.3	0.69	8.9	0.49	9.9	0.44	n.c.	n.c.	8.6	0.35
Sr	341.7	21.9	287.2	15.4	263.0	17.6	33.3	< 0.001*	314.9	18.8
Th	9.7	0.33	10.8	0.37	9.5	0.18	28.4	< 0.001*	9.6	0.27
Ti	3578.5	414.3	3118.5	87.7	4143.7	210.8	30.2	< 0.001*	3252.9	146.6
U	21.4	4.2	20.5	4.1	20.0	3.3	n.c.	n.c.	18.9	3.6
V	67.8	7.5	67.5	3.8	72.1	3.7	n.c.	n.c.	59.5	2.5
Y	13.6	0.61	15.0	0.55	14.1	0.57	n.c.	n.c.	13.2	0.40
Yb	1.5	0.09	1.6	0.06	1.6	0.04	n.c.	n.c.	1.5	0.07
Zn	61.6	4.1	56.6	3.4	62.7	1.4	n.c.	n.c.	53.4	2.3
Zr	113.7	10.1	110.7	4.8	119.5	5.9	n.c.	n.c.	106.4	4.7
Al/Ti	9.7	1.4	12.9	0.86	9.3	0.56	27.5	< 0.001*	10.7	0.57
Cu/Pb	2.9	0.54	2.6	0.24	2.7	0.26	n.c.	n.c.	2.9	0.45
Fe/Mn	48.0	2.6	36.7	4.1	50.5	1.7	n.c.	n.c.	50.7	2.5
Fe/P	36.6	4.5	40.3	2.5	37.0	4.0	n.c.	n.c.	34.1	1.4
Pb/Ni	0.05	0.01	0.13	0.02	0.07	0.01	32.6	< 0.001*	0.1	0.01
(K + Na + Ca + Mg)/Al	4.6	0.42	3.5	0.32	3.7	0.31	28.8	< 0.001*	4.3	0.28
K/Na	2.1	0.23	2.7	0.30	2.5	0.19	23.4	< 0.001*	2.6	0.31
PIA	21.0	1.3	25.1	1.8	25.5	1.8	26.5	< 0.001*	22.1	1.3
CIW	26.7	1.7	32.4	2.4	31.7	1.9	26.9	< 0.001*	28.1	1.7
CIA	25.5	1.6	30.6	2.1	30.2	1.8	26.6	< 0.001*	26.8	1.5
WIP	41.2	1.2	38.7	1.0	37.3	1.2	28.8	< 0.001*	38.9	0.93
Recycling	0.62	0.05	0.79	0.07	0.81	0.07	27.9	< 0.001*	0.69	0.06

^⁎^ Critical p-value = 0.05; n.c., non-conservative tracer.

The weathering indices were computed based on the molecular proportion of associated elements. Here, the concentration of elements (in ppm) was converted to elemental oxides based on each element compound ratio (Guo et al., 2018; Motha et al., 2003; Olley and Caitcheon, 2000). The Chemical Index of Alteration (CIA), originally proposed by Nesbitt and Young (1982) and applied by numerous studies (e.g., Guo et al., 2018; Shao et al., 2012), is potentially useful as a tracer since it provides a basis for identifying chemical changes caused by weathering of alumino-silicate minerals (Haddadchi et al., 2013; Motha et al., 2003; Owens et al., 2016). It is defined as ${\text{A}\text{l}}_{2}{\text{O}}_{3}/({\text{A}\text{l}}_{2}{\text{O}}_{3}\;+\;{\text{C}\text{a}\text{O}}^{\;}+\;{\text{N}\text{a}}_{2}\text{O}\;+\;{\text{K}}_{2}\text{O})\;\times 100$ (Guo et al., 2018; Olley and Caitcheon, 2000; Shao et al., 2012). A CIA value of approximately 45–55 shows a lack of weathering, whereas a value of 100 indicates intense weathering with the complete removal of alkali and alkaline earth elements from the parent material (Motha et al., 2003; Shao et al., 2012). In general, higher CIA values ​​suggest more weathering of silicates; however, it is difficult to make a simple linear relationship between weathering and CIA values (Shao et al., 2012). The WIP was proposed by Parker (1970) to evaluate the weathering intensity of silicate rocks based on the ratios of alkali and alkaline earth elements (Price and Velbel, 2003), and it can be defined as $(2{\text{N}\text{a}}_{2}\text{O}/0.35\;+\;\text{M}\text{g}\text{O}/0.9\;+\;2K_{2}\text{O}/0.25\;+\;\text{C}\text{a}\text{O}/0.7)\;\times \;100\;$ (Guo et al., 2018; Shao et al., 2012). According to the definition of WIP, and contrary to CIA values, smaller WIP values are indicative of stronger chemical weathering (Guo et al., 2017). This index is most suitable for weathering profiles on heterogeneous parent rocks but potentially less suitable for highly weathering coatings, because its formulation is based on only highly mobile alkali and alkaline elements (Hamdan and Bumham, 1996; Price and Velbel, 2003). In the case of the mobility of aluminum during chemical washing, some studies have shown that WIP may be more suitable for characterizing weathering intensity than CIA, although both indicators are well correlated. However, to date, WIP has been rarely used to measure the weathering intensity in different stream sediment samples from around the world, whereas CIA has been widely documented for drainage basins (Shao et al., 2012).

CIA-WIP plots can be used to discriminate the modification of sediment compositions due to weathering induced by recycling over longer timescales. Thus, with an increase in sediment recycling, the value of WIP is linearly reduced, but CIA is largely independent of this (Guo et al., 2017). The effects of chemical recycling on river sediment chemistry should be carefully considered in order to provide stronger constraints on the interpretation of sediment-associated geochemical profiles (Guo et al., 2018).

The Chemical Index of Weathering (CIW) developed by Harnois (1988) is defined as ${\text{A}\text{l}}_{2}{\text{O}}_{3}/({\text{A}\text{l}}_{2}{\text{O}}_{3}+{\text{C}\text{a}\text{O}}^{\;}+\;{\text{N}\text{a}}_{2}\text{O})\;\times 100$. This index is equivalent to the CIA, except that the potassium was eliminated from the CIA equation. Like the CIA, the CIW is also essentially a measure of the extent of conversion of feldspars to clays. Because the CIW does not account for the aluminum associated with K-feldspar, it may yield very high values for K-feldspar rich rocks, whether they are chemically weathered or not. Fedo et al. (1995) proposed the Plagioclase Index of Alteration (PIA) as an alternative to the CIW. Because plagioclase is abundant in silicate rocks and dissolves relatively rapidly, the PIA may be used when plagioclase weathering needs to be taken into consideration (Price and Velbel, 2003).

### 2.4 Tracer conservation tests

Sediment tracers should exhibit conservative behavior during sediment detachment and transport through a catchment (Haddadchi et al., 2015; Lamba et al., 2015). Accordingly, the first step in addressing this requirement is removal of non-conservative tracers from further analysis. Non-conservative tracers were identified and eliminated using three methods (Haddadchi et al., 2015; Nosrati et al., 2018b). Initially, the standard bracket or range test was performed to ensure that the tracer concentrations of the individual main stem target sediment samples (which were collected from the outlet of the main basin) were within the concentration range of the sub-basin source groups (Mukundan et al., 2010; Navratil et al., 2012; Nosrati et al., 2018b; Tiecher et al., 2018). At the next stage, the mean concentration of each tracer in each main stem target sediment sample was compared with the corresponding mean concentration range in the sub-basin samples (Haddadchi et al., 2015; Wilkinson et al., 2013). Again, the tracers that were not in the mean concentration range of the sub-basin spatial source bed sediment samples were removed from further analysis. These range tests do not confirm the complete absence of changes to tracer properties, but instead provide an initial screening for the removal of tracers that show significant alteration between upstream sampled sources and downstream sites for the collection of target sediment samples. Lastly, biplots of tracer pairings selected in the final composite signatures were used to assess the degree to which the relationships between tracers judged to be generally conservative, by the first two tests, were consistent between upstream and downstream bed sediment sampling sites.

### 2.5 Discrimination of the tributary sub-basin spatial sediment sources

Applying statistical tests is one widely used means of identifying sets of tracers that can discriminate robustly between potential sediment sources (Haddadchi et al., 2014; Nosrati et al., 2011). Three statistical methods were used to provide composite fingerprints for discriminating the three sub-basin spatial sources: (1) a combination of KW-H and discriminant function analysis (DFA), (2) a combination of KW-H and data mining analysis, and (3) a combination of KW-H and principal components & classification analysis (PCCA).

KW-H or one-way ANOVA is a nonparametric test that compares more than two groups, and tests the null hypothesis that different groups in comparison comprise a similar distribution or distributions with equal means (Collins and Walling, 2002, 2007; Smith and Blake, 2014). Therefore, the KW—H test helps eliminate any tracer that does not show significant differences in the concentrations between at least two sediment sources (Pulley et al., 2015).

Discriminant function analysis (DFA) was performed based on minimizing the Wilks’ lambda of those tracer properties that passed the KW-H test. DFA has been widely used to determine the final composite fingerprints that have the highest discrimination between sediment sources but with a minimum number of variables (Collins et al., 1998; Evrard et al., 2011; Lamba et al., 2015; Smith and Blake, 2014). Wilks’ lambda is a multivariate statistical test that measures the significance of the discriminatory power of the model. Wilks’ lambda values ​​are between 0-1. Values close to 0 ​​indicate high discriminatory power, while values ​​close to 1 indicate poor discrimination (Hughes et al., 2009). To determine whether discriminant functions are statistically significant, various tests including eigenvalue, canonical correlation and Mahalanobis distance were used (Nosrati, 2017; Nosrati et al., 2018b).

Tracers exhibiting statistically significant differences between the potential sub-basin spatial sediment sources, using KW—H, were also entered into classification tree analysis. Classification tree analysis is one of the main techniques used in data mining (Lemon et al., 2003). Classification trees are used to predict membership of cases in the classes of a categorical dependent variable from their measurements on one or more predictor variables (i.e. tracers) and as such, this method has much in common with DFA. Classification tree analysis determines a set of logical if-then conditions (instead of the linear equations estimated in DFA) for classifying cases. Classification trees readily lend themselves to being displayed graphically, helping to make them easier to interpret. There are two key tools to interpret the results of classification tree analysis: the final trees and the predictor importance. The final graphical sequence of (boosted) trees can be a useful way to examine the significance of the final classification model. The predictor importance is computed as the relative (scaled) average value of the predictor statistic over all trees. The bar plot of the predictor importance provides insight on the variables that make the major contributions to the prediction of group membership. The fourth and final set of tracer properties was identified by applying a PCCA test to those tracers that passed the KW—H test. A comprehensive review of the principal component method is presented by Nosrati et al. (2018a). All statistical analyses were performed using STATISTICA V.8.0 (StatSoft, 2008).

### 2.6 Source apportionment using a sediment un-mixing model

The Modified MixSIR Bayesian un-mixing model (Nosrati et al., 2014, 2018b) quantifies the relative contributions of sediment from different sources by calculating probability distributions for the proportional contribution (f_i_) of each source i to the downstream target sediment samples in three stages: 1) prior probability distributions for model parameters, 2) construction of a likelihood function for the statistical model, and 3) derivation of the posterior probability distributions for the parameters using the Bayes rule to adjust the prior distributions based on the observed data. The Bayes rule states that the posterior probability distribution for all f_i_ is proportional to the prior probability distributions multiplied by the likelihood, and then dividing by their sum, viz.:

(1) $$P(f_{q}\left|data)=\frac{L(data\left|f_{q})\times p(f_{q})\right)}{\sum L(data\left|f_{q})\times p(f_{q})\right)}\right)$$

where L(data|fq) is the likelihood of the data given f_q_, p(f_q_) representing the prior probability being true, based on prior information, and f_q_ is the proportional source contributions of q proposed vectors.

The relative contributions of sediment are factored into the model by defining mean and variance parameters for each sediment source i and the final sets of tracers (composite fingerprints; j).

The proposed tracer distributions for the target sediment mixtures collected from the study catchment outlet are determined by solving for the proposed means ${\hat{\mu }}_{j}$and standard deviations ${\hat{\sigma }}_{j}$of the sediment mixtures based on the randomly drawn f_i_ values comprising a vector f_q_:

(2) $${\hat{\mu }}_{j}=\sum_{i=1}^{n}\left(f_{i}\times m_{j_{Source_{i}}}\right)$$

(3) $${\hat{\sigma }}_{j}=\sqrt{\sum_{i=1}^{n}\left(f_{i}^{2}\times S_{j_{Source_{i}}}^{2}\right)}$$

where $m_{j_{Source_{i}}}$in Eq. (3) is the mean and $S_{j_{Source_{i}}}^{2}$ in Eq. (4) is the variance of the jth sediment tracer and the ith sediment source.

Based on the ${\hat{\mu }}_{j}$and ${\hat{\sigma }}_{j}$of each property comprising each final composite fingerprint, the likelihood of the data given the proposed sediment mixture is calculated as:

(4) $$L(x\left|{\hat{\mu }}_{j}\right),{\hat{\sigma }}_{j})=\prod_{k=1}^{n}\prod_{j=1}^{n}\left[\frac{1}{{\hat{\sigma }}_{j}\times \sqrt{2\times \pi }}\times \exp\left(-\frac{{\left(X_{kj}-{\hat{\mu }}_{j}\right)}^{2}}{2\times {\hat{\sigma }}_{j}^{2}}\right)\right]$$

where $X_{kj}$represents the *j*^th^ tracer property of the *k*^th^ sediment sample.

The model predictions of source proportions were evaluated using 10 sets of virtual sediment mixtures for each composite signature using a range of source proportions: 1) equal proportions from each sub-basin – 33.3%, 33.3%, 33.3%; 2) 100% sub-basin 1, 0% sub-basins 2, 3; 3) 0% sub-basin 1, 100% sub-basin 2, 0% sub-basin 3; 4) 0% sub-basins 1, 2, 100% sub-basin 3; 5) 50% sub-basin 1, 25% sub-basin 2, 25% sub-basin 3; 6) 25% sub-basin 1, 50% sub-basin 2, 25% sub-basin 3; 7) 25% sub-basin 1, 25% sub-basin 2, 50% sub-basin 3; 8) 75% sub-basin 1, 10% sub-basin 2, 15% sub-basin 3; 9) 15% sub-basin 1, 75% sub-basin 2, 10% sub-basin 3, and; 10) 10% sub-basin 1, 15% sub-basin 2, 75% sub-basin 3. Since the virtual sediment mixtures were constructed using the measured tracer data for the tributary sub-basin spatial source samples, the tracer concentrations in the virtual mixtures satisfied the bracket test for tracer conservation. The accuracy of the modelling in solving the virtual sediment mixtures was assessed using the averaged root mean square error (RMSE) and mean absolute error (MAE) between the predicted and known source proportions using each final composite signature.

## 3 Results and discussion

### 3.1 Confirmation of conservative tracers and different composite fingerprints for discriminating the spatial sediment sources

Table 1 compares the tracer concentrations in the sub-basin outlet (spatial sediment sources) and downstream target sediment samples. The results of the standard bracket test showed that Ag, Ce, Cu, La, Mn, Pb, Sb, Sc, U, V, Yb, Zn, Zr, Cu/Pb and Fe/Mn were non-conservative. In addition to the standard test, the results of comparing the sediment means with the corresponding sub-basin spatial source means showed that all remaining tracers, except Y and Fe/P, were conservative (Table 1). On the basis of these two tests, the 29 remaining tracers were retained and tested using the KW-H test. Table 1 also shows the results of applying the KW-H test which indicated that all tracers exhibited a statistically significant difference between the tributary sub-basin spatial sediment sources using the bed sediment samples collected from the field.

The 29 tracers (Al, As, Ba, Be, Ca, Cd, Co, Cr, Fe, K, Li, Mg, Mo, Na, Ni, P, S, Sr, Th, Ti, Al/Ti, K/Na, Pb/Ni, (K + Na + Ca + Mg)/Al, CIA, WIP, Recycling, CIW and PIA) passing the KW—H test were entered into the stepwise DFA (Table 2). The largest eigenvalue of the first function (61.1) corresponds to the eigenvector in the direction of the maximum spread of the groups’ means. The Wilk’s lambda value of the first function (0.001) indicated that 83% of the total variance among the sub-basin spatial source bed sediment samples was explained by these tracers. The canonical correlation value was 0.99 and indicated a strong correlation between the discriminant scores and the individual source groups (Table 3). The squared Mahalanobis distance showed that the spatial sediment sources were well separated by the shortlisted tracers (Table 3). The backward stepwise DFA yielded classification matrices assigning 100% of the cases (i.e., spatial source bed sediment samples) to the correct groups (Table 3).

**Table 2:** Summary of the backward Discriminant Function Analysis (DFA).

Tracer	Wilks' lambda	Partial Wilks' lambda	F-remove	p-level	Tolerance
Li	0.01	0.24	52.6	0.00	0.83
Al	0.01	0.13	109.1	0.00	0.94
Ba	0.00	0.42	22.9	0.00	0.64
P	0.01	0.31	36.9	0.00	0.53
Al/Ti	0.01	0.37	28.7	0.00	0.93
Na	0.00	0.53	14.7	0.00	0.64
Fe	0.00	0.38	27.1	0.00	0.85

**Table 3:** Summary of the backward Discriminant Function Analysis (DFA) using the composite sediment samples collected from each sub basin.

DFA parameters	Output
Function 1	
Eigenvalue	61.1
Wilks' lambda	0.001
Canonical correlation	0.99
Function 2	
Eigen value	19.5
Wilks' lambda	0.049
Canonical correlation	0.98
Spatial sediment sources samples classiﬁed correctly (%)	
Sub-basin 1	100
Sub-basin 2	100
Sub-basin 3	100
Total	100
Squared Mahalanobis distance	
Sub-basin 1 × Sub-basin 2	79.3
Sub-basin 1 × Sub-basin 3	155.8
Sub-basin 2 × Sub-basin 3	209.5
Squared Mahalanobis F-value	
Sub-basin 1 × Sub-basin 2	75.5^a^
Sub-basin 1 × Sub-basin 3	121.1^a^
Sub-basin 2 × Sub-basin 3	147.8^a^

a Significant at 0.01 level.

**Fig. 3:**
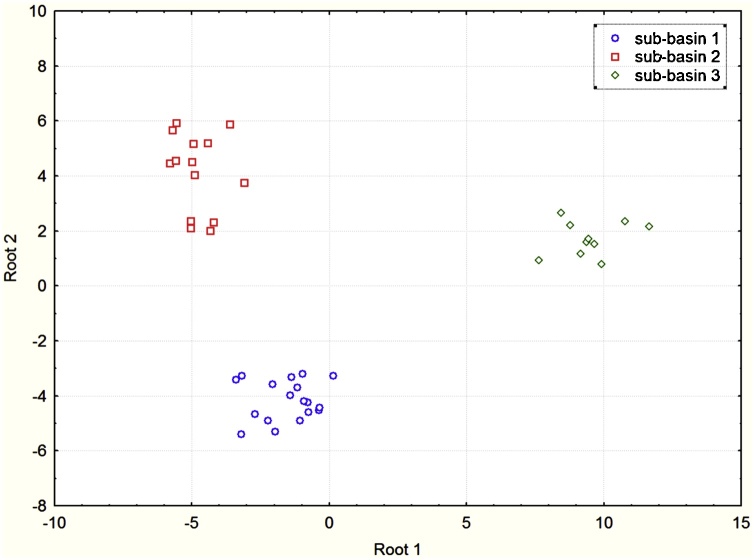
Scatterplot of the first and second discriminant functions calculated using backward DFA associated with selection of the composite signature comprising Al, Ba, Fe, Li, Na, P, and Al/Ti.

Stepwise selection using Wilks' lambda indicated that a composite signature comprising seven tracers (Li, Al, Ba, P, Al/Ti, Na and Fe) provided significant discriminatory power on the basis of the DFA model (Table 2). The results of different tests within DFA indicated that the discriminatory power of all of these tracers was perfect (Table 2). Partial Wilks' lambda is the Wilks' lambda for the unique contribution of the respective tracer to the discrimination between individual source groups. The smaller the Partial Wilks' lambda, the greater the contribution to the overall discrimination. The Partial Wilks' lambda values suggested that Al contributed the most, Li second most, P third most, Al/Ti fourth most, Fe fifth most, Ba sixth most and Na the least to the overall discrimination (Table 2). A scatterplot (Fig. 3) using the first and second discriminant functions calculated using backward DFA confirmed that the sediment samples collected from the outlets of the different sub-basin spatial sediment sources were well separated.

**Fig. 4:**
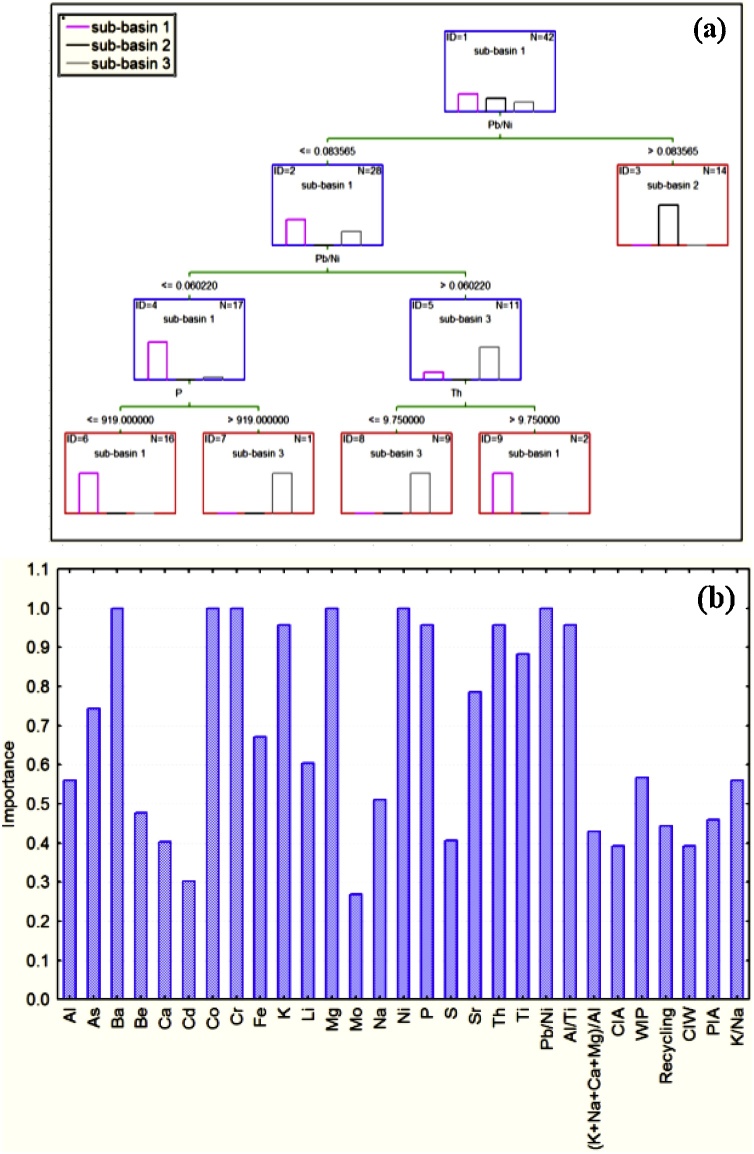
(a) The classification trees for the sub-basin spatial sediment sources, and (b) importance plots for the sub-basin spatial sediment sources.

During the data mining stage of the tracer analysis, the properties passing KW-H were entered into general classification and regression tree models (GC&RT). Fig. 4a displays the final tree selected on the basis of the sub-basin properties (tracers) considering v-fold cross-validation (CV cost = .03) analysis. The final tree classified the sub-basin spatial sediment sources into three terminal nodes (i.e., the sampled sub-basins) using the sediment samples collected to characterise these potential sources. The histograms of cases in each class at the nodes showed that 100% of the cases were classified correctly (Fig. 4a).

The bar plot of predictor importance based on tributary sub-basin spatial source as the dependent variable and the tracer properties as the predictors is presented in Fig. 4b. For each tracer, predictor importance shows the relative average value of the sums-of-squares prediction over all trees and nodes, where the maximum value sums to 1. Hence, these values reflect the strength of the relationship between the predictors and the dependent variable of interest over the successive boosting steps. In this case, the tracers Ba, Co, Cr, Mg, Ni, and Pb/Ni (importance value = 1.0) stand out as the most important predictors within this particular composite signature (Fig. 4b). These results provide evidence that general classification and regression tree models can be used as a tool for identifying important dimensions in a set of tracers included within a composite signature and to identify those sediment sources with similar or dissimilar characteristics.

Tracers passing the KW—H test (Al, As, Ba, Be, Ca, Cd, Co, Cr, Fe, K, Li, Mg, Mo, Na, Ni, P, S, Sr, Th, Ti, Al/Ti, K/Na, Pb/Ni, (K + Na + Ca + Mg)/Al, CIA, WIP, Recycling, CIW and PIA) were also tested using PCCA. Here, all the tracers in question were further explored as an alternative means of reducing the number of tracers and problems of multicollinearity. The results of PCCA showed that the first five principal components (PCs) with eigenvalues >1 accounted for >90% of the variability among the tracer values for the three spatial source groups (Table 4). The PC corresponding to the largest eigenvalue (16.0) accounted for approximately 55% of the total variance. The second PC corresponding to the second eigenvalue (5.9) accounted for approximately 20% of the total variance. The third PC corresponding to the third eigenvalue (1.9) accounted for approximately 6% of the total variance. The fourth PC corresponding to the fourth eigenvalue (1.1) accounted for approximately 4% of the total variance. The PC corresponding to the smallest selected eigenvalue (1.0) accounted for approximately 3.6% of the total variance (Table 4).

**Table 4:** Principal component and classification analysis (PCCA) factor coordinates of the variables and the eigenvalues of the correlation matrix.

Tracer	PC 1	PC 2	PC 3	PC 4	PC 5	Communalities
Al	−0.93	−0.22	0.20	−0.08	0.05	0.87
As	−0.73	0.08	0.02	−0.10	0.03	0.54
Ba	−0.81	0.43	−0.10	−0.27	0.03	0.66
Be	**-0.93**^a^	0.06	0.18	−0.12	0.10	0.87
Ca	0.70	0.62	0.13	0.10	−0.05	0.49
Cd	−0.38	0.20	−0.56	0.31	0.47	0.15
Co	0.83	−0.41	0.05	−0.26	0.12	0.68
Cr	0.73	−0.12	−0.32	**-0.44**	0.07	0.54
Fe	0.45	−0.81	0.14	−0.26	0.12	0.20
K	−0.89	0.36	0.15	−0.10	0.08	0.80
Li	−0.34	0.53	**0.66**	−0.11	0.30	0.11
Mg	0.89	−0.26	0.18	−0.20	0.18	0.79
Mo	−0.59	0.16	−0.39	0.19	0.18	0.34
Na	0.41	0.62	−0.27	−0.24	−0.18	0.17
Ni	0.90	−0.25	0.09	−0.19	0.13	0.81
P	0.56	−0.39	0.31	**0.47**	0.25	0.32
S	0.44	0.08	−0.38	−0.19	**0.63**	0.20
Sr	0.53	0.70	0.37	−0.08	0.18	0.28
Th	−0.81	0.39	−0.04	−0.28	−0.01	0.66
Ti	0.54	**-0.76**	−0.03	−0.08	−0.09	0.29
Al/Ti	−0.87	0.42	0.07	−0.04	0.09	0.76
K/Na	−0.89	−0.15	0.24	0.06	0.15	0.79
Pb/Ni	−0.86	0.21	−0.31	−0.17	−0.14	0.74
(K + Na + Ca + Mg)/Al	0.93	0.33	−0.08	−0.02	−0.01	0.86
CIW	−0.88	−0.44	0.06	−0.10	0.05	0.78
PIA	−0.81	−0.56	0.07	−0.08	0.04	0.66
CIA	−0.87	−0.46	0.07	−0.09	0.05	0.76
WIP	0.54	**0.77**	0.22	−0.07	0.03	0.29
Recycling	−0.79	−0.60	−0.02	−0.04	0.02	0.63
Eigenvalue	16.0	5.9	1.9	1.1	1.0	
% Total variance	55.3	20.2	6.5	3.9	3.6	
Cumulative % variance	55.3	75.5	82.0	85.9	89.5	
Mean scores of the three spatial sediment sources						
Sub-basin 1	0.88a^b^	0.48a	0.29a	−0.17a	0.17a	
Sub-basin 2	−1.12b	0.43b	−0.43ab	−0.32ab	−0.13a	
Sub-basin 3	−0.01c	−1.47c	0.08b	0.76c	−0.11a	
ANOVA results						
F-value	33.8	21.8	7.2	8.3	0.20	
p-Value	0.00	0.00	0.03	0.02	0.90	

a The highly-weighted tracers selected as an alternative composite fingerprint.

b Different small letters indicate that scores are significantly different at the 0.05 level of confidence, based on the Tukey HSD Post Hoc test.

**Fig. 5:**
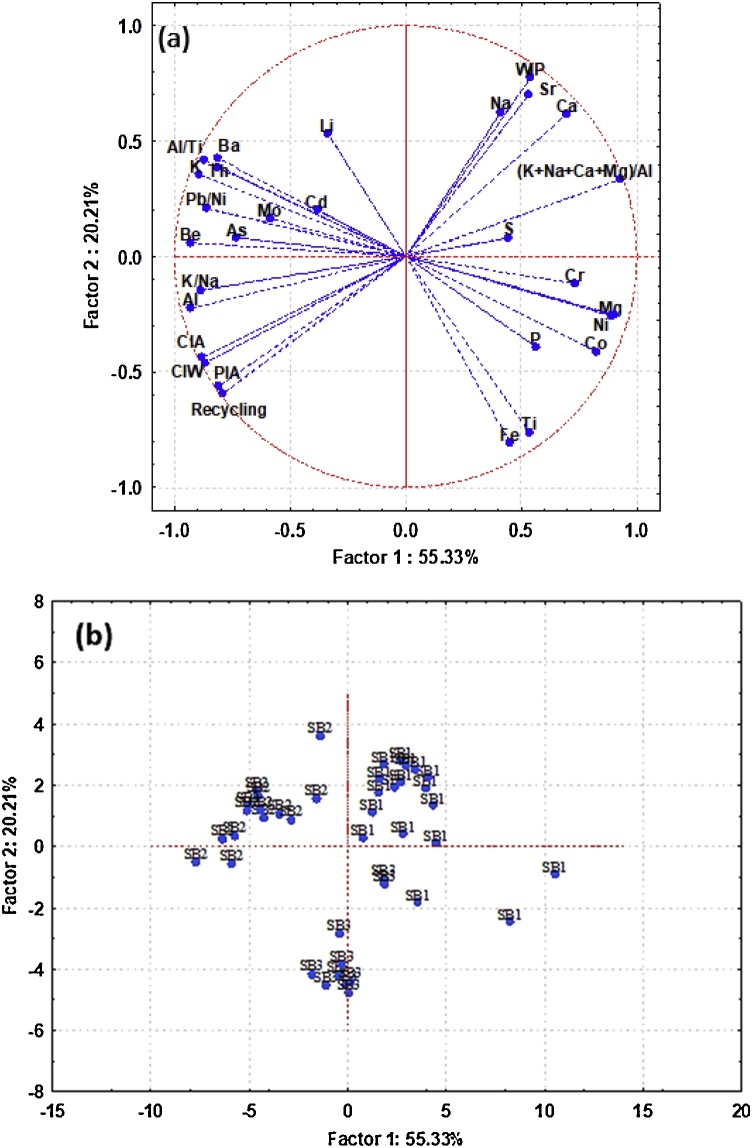
(a) Projection of the final composite fingerprint on the factor-plane using PCCA, (b) Projection of the cases on the factor-plane using PCCA; SB 1: sub-basin 1; SB 2: sub-basin 2; SB 3: sub-basin 3.

The highly-weighted tracers under PC1 with absolute values within 10% of the highest tracer (0.93 value for Be) loading (the loading of selected tracers should be larger than 0.84) were Al, K, Mg, Ni, Pb/Ni, Al/Ti, K/Na, (K + Na + Ca + Mg)/Al, CIA and CIW. Only Be was retained for the final composite signature because these eleven tracers were strongly inter-correlated. Under PC2, the highly-weighted tracers with absolute values within 10% of the highest tracer (0.77 value for WIP) loading (the loading of selected tracers should be greater than 0.70) were Sr and Ti. Only WIP and Ti were retained for the final composite signature because WIP was strongly correlated with Sr (r = 0.92). Under PC3, the highly-weighted tracer (0.66 value for Li) with absolute values within 10% of the highest tracer loading (the loading of selected tracers should exceed 0.60) was Li. Under PC4, the highly-weighted tracers with absolute values within 10% of the highest tracer (0.47 value for P) loading (the loading of selected tracers should exceed 0.42) were P and Cr. Both were retained for the final composite signature because they were not strongly correlated. Under PC5, the highly-weighted tracer (0.63 value for S) with absolute values within 10% of the highest tracer loading (the loading of selected tracers should exceed 0.57) was S. PCs scores were calculated using the resulting component score coefficient matrix and tested for significant differences between the sediment sources using one-way ANOVA (F-test) and Tukey HSD post-hoc tests (P < 0.05) (Table 4). The results showed that the PC scores for the first four PCs varied significantly with sediment sources. So the tracer associated to the PC5 (S) was excluded from the composite signature. These results thereby selected six tracers (Be, Fe, WIP, Li, P and Cr) as an alternative composite fingerprint on the basis of the PCCA model (Table 4). The plot of factor coordinates of variables for the first two PCs associated with the six tracers selected by PCCA is presented in Fig. 5a. This composite fingerprint clearly provided strong discrimination between the three potential sub-basin sediment sources (Fig. 5b).

**Fig. 6:**
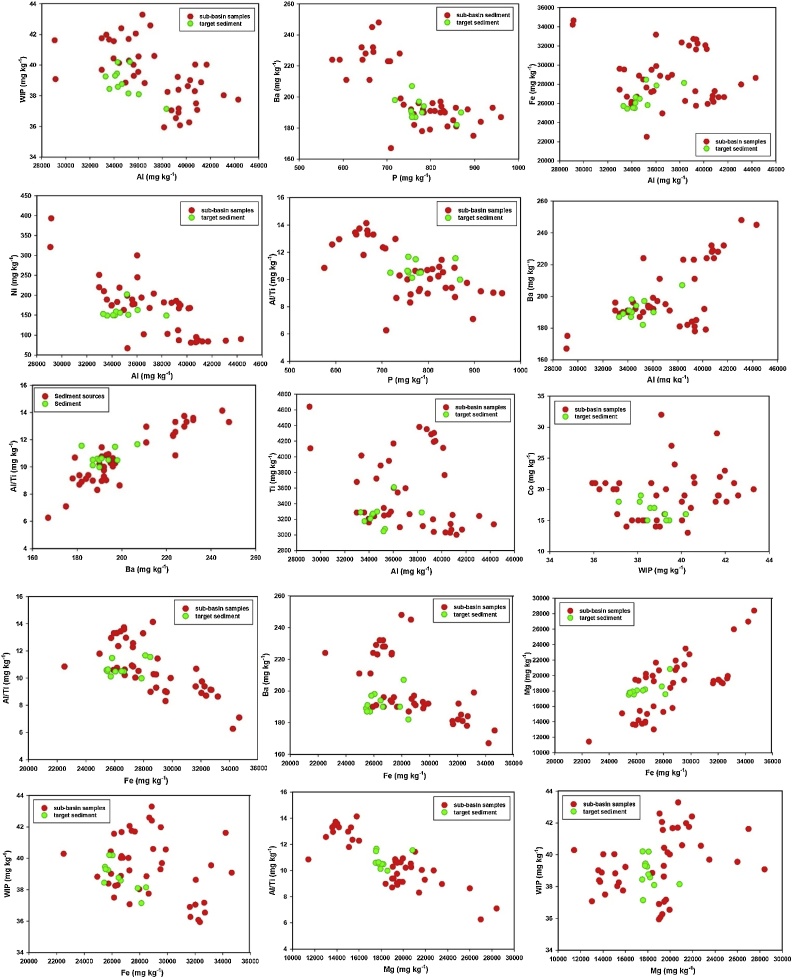

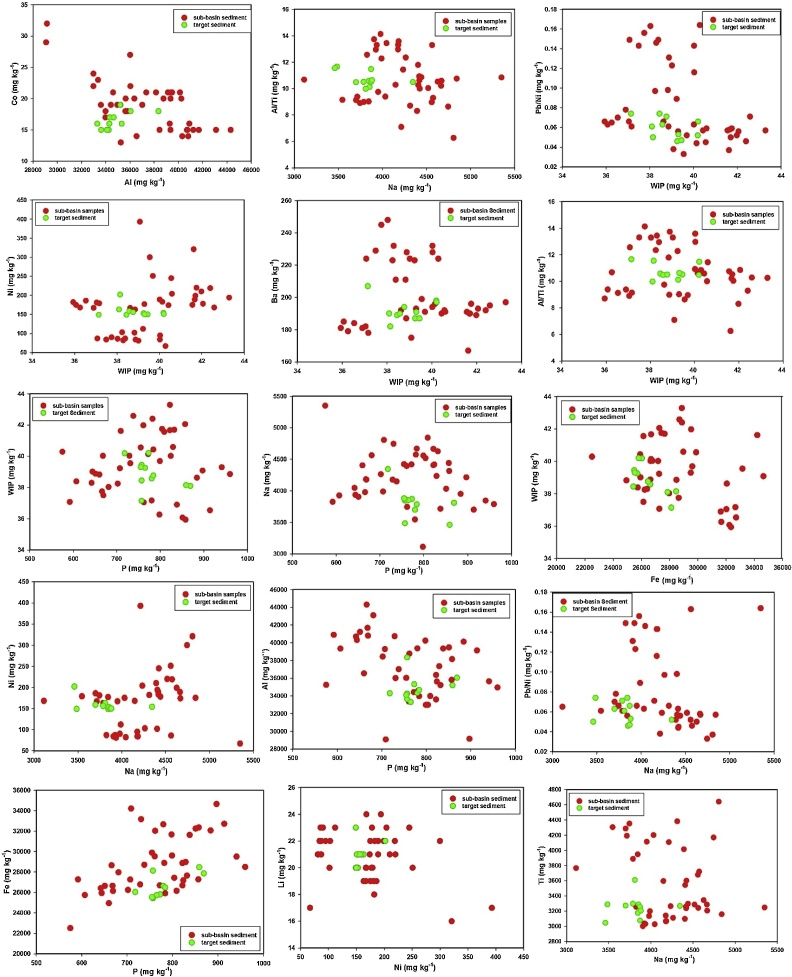
Biplots of some pairings for the tracers selected in the final composite signatures for discriminating and apportioning source contributions to target sediment samples.

For the tracers selected in the final composite signatures, the biplots of some tracer pairings for source and sediment samples were compared. The results confirmed that there was no major tracer transformations during sediment mobilization and delivery in the study area (Fig. 6).

### 3.2 Sediment source contributions

**Fig. 7:**
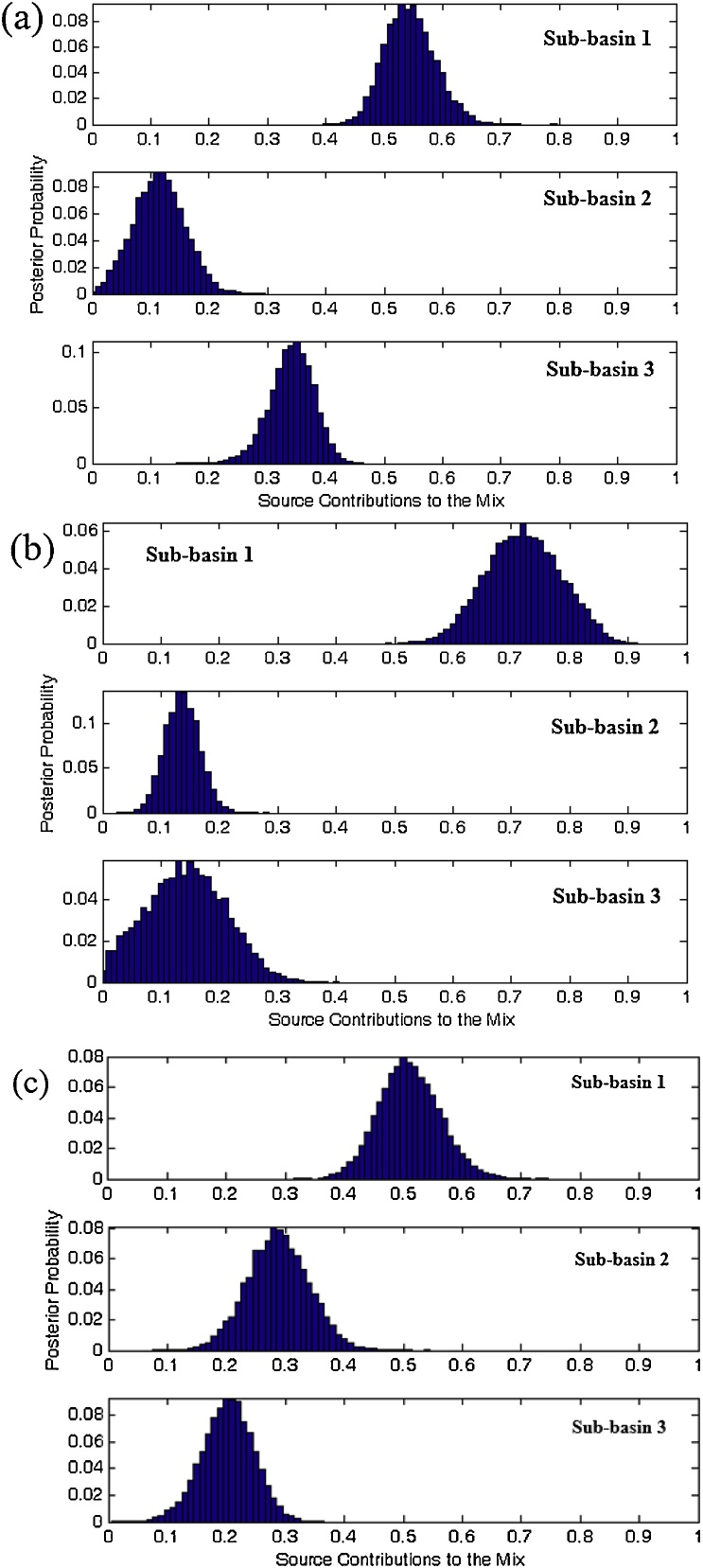
Posterior probability density functions for the estimated sub-basin spatial sediment source contributions using the final composite signatures selected by (a) a combination of KW-H and discriminant function analysis (DFA), (b) a combination of KW-H and data mining analysis, and (c) a combination of KW-H and principal components & classification analysis (PCCA).

Fig. 7 presents the un-mixing model outputs. Using the composite fingerprint selected by KW-H and DFA (Table 5), the relative contributions (with corresponding uncertainty ranges) from the sub-basin spatial sources 1, 2 and 3 were estimated as 54.3% (47.8–62.0), 11.4% (4.2–18.7), and 34.3% (27.6–39.9), respectively. Using the alternative composite signature selected by a combination of KW-H and data mining (Table 5), the corresponding respective contributions and associated uncertainty ranges were estimated as 72.0% (61.6–82.7), 13.6% (9.0–18.5) and 14.2% (3.1–25.4). Finally, on the basis of the composite signature selected using a combination of KW-H and PCCA (Table 5), the relative contributions from the sub-basin spatial sources 1, 2 and 3 were estimated as 50.8% (42.8–59.9), 28.7% (20.2–37.3) and 20.3% (12.7–27.2), respectively. The root mean square difference (Table 5) between the estimated sediment contributions from the sub-basin spatial sources using the three different composite signatures ranged from 22.3% (sub-basin 3) to 47.4% (sub-basin 2). The predicted spatial source contributions were therefore highly sensitive to the composite fingerprint used in the Bayesian un-mixing model.

**Table 5:** Source apportionment results using the final composite signatures and corresponding root mean square differences between the results.

Statistical approaches for selecting final composite signatures	Spatial sediment source		
	Sub-basin 1 (%)	Sub-basin 2 (%)	Sub-basin 3 (%)
Combination of KW-H and DFA (Tracers: Al, Ba, Fe, L i, Na, P, Al/Ti)	54.3 (47.8-62.0)^a^	11.4 (4.2-18.7)	34.3 (27.6-39.9)
Combination of KW-H and data mining (Tracers: Ba, Co, Cr, Mg, Ni, Pb/Ni)	72.0 (61.6-82.7)	13.6 (9.0-18.5)	14.2 (3.1-25.4)
Combination of KW-H and PCCA (Tracers: Be, Cr, Li, P, Ti, WIP)	50.8 (42.8-59.9)	28.7 (20.2-37.3)	20.3 (12.7-27.2)
Root mean square difference	30.4	47.4	22.3

a The values in parentheses show the uncertainty ranges (90% confidence limits: 5%–95%).

Comparisons of the predicted and known relative contributions from the tributary sub-basin spatial sources 1, 2 and 3 using the three different composite signatures and the virtual sediment mixtures showed that the RMSE ranged between 0.6% and 35.8% and MAE between 0.2% and 11.9% (Table 6). The overall average RMSE and MAE for the modelled source predictions using the virtual mixtures were 10.6% and 9.6%, respectively. The RMSE results showed that 15 of 30 possible predicted values were < 10%, whilst in the case of MAE, 29 of the 30 values were < 10%. These error levels were judged to be acceptable in the context of those reported elsewhere in the international literature (e.g., Haddadchi et al., 2013; Nosrati and Collins, 2019; Palazón et al., 2015).

**Table 6:** Comparison of the predicted and known relative contributions from the sediment sources to the virtual sediment mixtures using the composite signatures selected by different statistical approaches and the corresponding root mean square error (RMSE) and mean absolute error (MAE).

Statistical approaches for selecting composite fingerprints	Known sediment source proportions			Predicted sediment source proportions			RMSE	MAE
	1	2	3	1	2	3		
Combination of KW-H and DFA (Tracers: Al, Ba, Fe, Li, Na, P, Al/Ti)	33.3	33.3	33.3	30.2	31.1	38.7	3.8	1.3
	100	0	0	71	9.8	17.6	20.4	6.8
	0	100	0	7.5	84.3	5.9	10.6	3.5
	0	0	100	11.5	4.5	83.1	12.1	4.0
	50	25	25	38.1	27.6	34.1	8.8	2.9
	25	50	25	22.9	48.1	28.1	2.4	0.8
	25	25	50	24.1	25.6	49.9	0.6	0.2
	75	10	15	55.8	15.7	27.8	13.7	4.6
	15	75	10	16	69	13.1	3.9	1.3
	10	15	75	17.1	14.7	67.2	6.1	2.0
Combination of KW-H and Data mining (Tracers: Ba, Co, Cr, Mg, Ni, Pb/Ni)	33.3	33.3	33.3	15.4	34.2	49.8	14.1	4.7
	100	0	0	61.3	29.4	7.8	28.4	9.5
	0	100	0	2.5	92	4.6	5.5	1.8
	0	0	100	23.9	26.3	49.2	35.8	11.9
	50	25	25	33.2	34.7	31	11.7	3.9
	25	50	25	10.7	45	43.8	13.9	4.6
	25	25	50	17.8	31.4	50.1	5.6	1.9
	75	10	15	51.6	32	14.6	18.5	6.2
	15	75	10	6.1	68.5	24.5	10.5	3.5
	10	15	75	14	26.9	59.3	11.6	3.9
Combination of KW-H and PCCA (Tracers: Be, Cr, Li, P, Ti, WIP)	33.3	33.3	33.3	23.8	36.5	38.6	6.5	2.2
	100	0	0	70.3	12.2	14.9	20.4	6.8
	0	100	0	4.2	90.6	4.1	6.4	2.1
	0	0	100	6	7.5	84.3	10.6	3.5
	50	25	25	38.6	29.2	31.5	8.0	2.7
	25	50	25	18.4	51.6	28.8	4.5	1.5
	25	25	50	21.7	25.2	51.7	2.1	0.7
	75	10	15	51.9	21.7	25.4	16.1	5.4
	15	75	10	12.6	72	14.1	3.2	1.1
	10	15	75	11.2	15.6	71.5	2.2	0.7

The weathering indices included in the initial potential tracer datasets reflect the intensity of chemical weathering processes and the degree of elemental mobility resulting from depletion of sediment samples in mobile as compared with immobile components (Nadlonek and Bojakowska, 2018; Price and Velbel, 2003). Only WIP was selected in one of the final composite signatures for discriminating the three tributary sub-basin spatial sources (Table 5, Table 6), although the results of the KW-H in Table 1 show that the indices were individually able to discriminate between the three sub-basin spatial sources. The WIP values in Table 1 suggest that the degree of chemical weathering reflected in the bed sediment samples collected to characterise sub-basins 2 and 3 is marginally greater than that for the equivalent samples collected from sub-basin 1. The estimates of WIP shown in Table 1 are considerably higher than those reported by some recent studies in the international literature (e.g., Nadlonek and Bojakowska, 2018) thereby suggesting comparatively lower chemical weathering intensity in the study area. The low values for CIA in Table 1 are also suggestive of a lack of intensive weathering and chemical alteration in the study area and reflect the local arid climate (Nadlonek and Bojakowska, 2018). Weathering index values reflect the complex interplay between climate, lithology, tectonism, topography, vegetation cover and human activities (Gibbs, 1970; Meybeck, 1987; Oliva et al., 2003; Shao et al., 2012). On this basis, they potentially offer a physically-grounded basis for inclusion in tracer datasets for identifying statistically robust composite signatures for source discrimination. Here, however, scale is likely to be an important factor driving the magnitude of differences between weathering index values for soil or sediment samples collected to characterise different river basin sources.

The source apportionment results based on the three composite signatures consistently suggested that tributary sub-basin 1 is the dominant source of the target sediment samples collected further downstream from the bed of the main stem. This sub-basin is the largest (168.3 km^2^) of the spatial zones apportioned in this study and comprises larger areas of both dry-land farming (72 km^2^) and rangelands (64 km^2^) compared to the other two spatial sources. Locally, dry-land farming is characterized by cultivation using conventional tillage practices on steep slopes, which renders bare tilled soils susceptible to rain splash detachment and subsequent mobilization in surface runoff. Recent expansion of such farming in sub-basin 1 on the Quaternary deposits has exacerbated erosion problems in this zone of the study area. At present, water scarcity problems and limited availability of new land for conversion to agriculture, mean that land use change from rangeland to arable cropping under dry-land farming is common, especially in sub-basin 1. Conventional dry-land farming practices are widespread, including intensive tillage with ploughing and heavy harrowing plus the routine removal of crop residues by burning to reduce biomass volume and to facilitate mechanical operations which are the primary factor leading to increased soil erosion and sediment production on the steep slopes (Jin et al., 2007). It should be noted however, that flood water irrigation in riparian areas in the lower and middle portions of sub-basin 1, which is assisted by siltation encouraging more overbank inundation, is used to support crop production on low lying riparian plots.

The source apportionment results presented herein should, however, be interpreted as a tier 1 assessment of the sources of downstream sediment issues. In this regard, they provide a basis for targeting follow-up work for confirming the relative losses from dry-land and rangeland farming practices, especially in sub-basin 1, to help engage local farmers and select and target appropriate mitigation measures for soil tillage or grazing management. Here, further work could also usefully assess the relative age of sampled sediment (e.g. using the ratios of fallout radionuclides with shorter and longer half-lives) to elucidate the likely temporal duration of sediment storage and remobilization in the study catchment and to help link the sediment source tracing results to oral cropping histories developed with local farmers.

## 4 Conclusions

A spatial sediment source fingerprinting exercise has been used to provide a tier 1 screening of sediment provenance in a mountainous agricultural catchment in western Iran. Three composite signatures were selected using different statistical tests, all of which suggested that sub-basin 1, draining the upper portions of the study area, dominates source contributions to the target sediment samples collected on the riverbed at the catchment outlet. The consistency of the signatures in all identifying sub-basin 1 as the dominant spatial sediment source lends weight to the results, since it is established that different composite signatures have the potential to generate contrasting sediment source estimates using mixing models (Collins et al., 2017b). The inclusion of weathering indices in initial tracer sets from which composite signatures are selected warrants further evaluation. Regardless of their inclusion or otherwise in final composite signatures, weathering indices potentially offer useful information for helping pre-selection of tracers (e.g. mineralogical, elemental) and for interpreting tracer conservation test results in the context of climate, lithology and hydro-geomorphological processes in river basins. With regards tracer conservation, longitudinal sediment sampling in large drainage basins would permit the weathering indices to be used to explore any potential evolution of tracer transformation risks across scales. Weathering indices offer potential for ensuring composite signatures have a physical, rather than a purely statistical basis, in the context of study area lithology and the requirement to discriminate individual spatial sources across landscapes. Follow-up investigations are required to confirm the relative contributions of the dry-land and rangeland farming, and of channel banks, in the study area to downstream sediment loads. Nonetheless, the evidence generated by this study will be used to help engage land owners in the need for regenerative farming practices.
